# Validity, Reliability, and Responsiveness of a New Device for Measuring Pressure Pain Thresholds

**DOI:** 10.3390/jcm15176687

**Published:** 2026-08-28

**Authors:** Rodrigo Martín-San Agustín, Javier Guerra-Armas, Alberto Gadea-Blázquez, Elena Millán-Magariños, Borja Tronchoni-Crespo, Iván José Fuentes-Abolafio, Adrian Escriche-Escuder

**Affiliations:** Clinimetry and Technological Development in Therapeutic Exercise Research Group (CLIDET), Department of Physiotherapy, University of Valencia, 46010 Valencia, Spain; rodrigo.martin@uv.es (R.M.-S.A.); gadea1610@gmail.com (A.G.-B.); e.millanmagarinos@edu.gva.es (E.M.-M.); borja.tronchoni@uv.es (B.T.-C.); adrian.escriche@uv.es (A.E.-E.)

**Keywords:** physiotherapy, digital health, measurement, musculoskeletal pain

## Abstract

**Background/Objectives** The evaluation of treatment effectiveness largely depends on the outcome measures used in the study. Patient-reported outcome measures (PROMs), such as the visual analog scale (VAS), and more objective measures, such as pressure pain thresholds (PPT), are commonly employed to capture pain-sensory profiles and hypoalgesic effects. The NOD is a novel digital algometer designed to assess the PPT. This study aimed to evaluate the validity, reliability, and responsiveness of the NOD algometer using a conventional algometer as a reference instrument for PPT assessment. **Methods**: A prospective longitudinal study with repeated measures was conducted following the COSMIN recommendations for studies evaluating measurement properties. Healthy participants with latent trigger points were recruited from the University of Valencia, where an experimental pain induction procedure was used to generate transient changes in PPT values and evaluate the responsiveness of the NOD device. The PPT was assessed at four time points: baseline, immediately after the intervention, and at 30 and 60 min post-intervention. **Results**: A total of 40 participants were included in the study (mean age: 22.0 ± 3.35 years; F = 35%). For concurrent validity, a strong positive association was observed between the reference algometer and NOD (Spearman’s ρ = 0.90, *p* < 0.001), and the limits of agreement ranged from −1.13 to 1.34 (−25.45% to 30.18%). For reliability, intra-session reliability was good to excellent in both sessions (ICC = 0.825 and 0.859), with a standard error of measurement (SEM = 0.63 to 0.82) and minimal detectable change (MDC = 1.75 to 2.26). For responsiveness, significant reductions in NOD values were observed at all post-intervention time points compared to baseline (*p* ≤ 0.005). The magnitude of change increased over time, with a standardized response mean (SRM = −0.57 to −0.74) and moderate-to-large effect sizes (Cohen’s d = 0.57–0.73). **Conclusions**: Our findings indicate that the NOD may be a valid and reliable tool for measuring PPT at latent trigger points in healthy participants. The NOD showed a strong association with a reference algometer and good relative reliability under standardized experimental conditions. The device was also able to detect experimentally induced changes in PPT. Nevertheless, further studies are required to establish its interchangeability with conventional algometers, responsiveness to clinically meaningful change, and applicability in clinical populations.

## 1. Introduction

Chronic pain currently affects 30% of the world’s population [1], causing economic, social, and personal harm [2]. Musculoskeletal pain is one of the most common forms of chronic pain [3]. Most people experience musculoskeletal pain at least once during their lifetime [3]. Therefore, musculoskeletal pain is a common and costly condition [3]. It is the second most common cause of disability in the general population [4]. Among musculoskeletal pain, myofascial pain syndrome (MPS) is one of the most common forms of chronic pain, with a lifetime prevalence of 85% in adults [5], comprising between 30% and 93% of individuals with musculoskeletal pain [6].

MPS is characterized by regional musculoskeletal pain, taut bands, tender points (trigger points), and referred pain patterns [7,8,9,10,11,12]. Active trigger points are associated with spontaneous pain and reproduction of the patient’s familiar pain, whereas latent trigger points are only painful to pressure but still limit movement and increase muscle tension [7,10,11,12,13]. The development and persistence of trigger points have been associated with factors such as muscle overuse, repetitive low-intensity contractions, trauma, and postural or occupational overload [7,11]. Several pathophysiological mechanisms have been proposed to explain their development and persistence, each supported by different empirical evidence [8,10,11,13,14,15]. Literature more consistently supports downstream phenomena than any single initiating lesion. Across reviews, the better-substantiated findings are abnormal electrical activity at motor endplates, altered local biochemical milieu, measurable stiffness or blood-flow differences on imaging, and links to peripheral or central sensitization [10,11,15]. By contrast, the causal chain that runs from excess acetylcholine to sustained sarcomere shortening to ischaemia to pain remains an integrated hypothesis rather than a settled mechanism [8,13,14]. Furthermore, systemic, psychological, and lifestyle factors, in addition to comorbidities, are believed to maintain or amplify myofascial pain syndrome [7,16]. However, the definition, aetiology, and detection of active and latent trigger points remain the subject of debate in the scientific literature [17,18].

An international Delphi panel proposed three essential criteria for a trigger point: taut band, tender point, and referred pain [9,10]. It was also agreed that the distinction between the active and latent phases is primarily based on the patient’s ability to reproduce and recognize symptoms [9]. However, comprehensive reviews have identified at least 19 distinct sets of criteria in the literature and considerable variability in their combinations, with many trials not clearly reporting their criteria [19,20]. Despite this, the diagnosis is based primarily on manual palpation of taut bands and trigger points, assessment of local and referred pain, contraction response, and muscle dysfunction (weakness, limited range of motion) [7,12,13]. Although the reliability of this method is considered acceptable, it is limited by subjectivity, inconsistent criteria, and lack of standardization [10,12,19], making the diagnosis predominantly clinical and subjective [20,21,22].

Several therapeutic approaches have demonstrated effectiveness in managing MPS, particularly in reducing pain [8,9,12,23]. However, the evaluation of treatment effectiveness largely depends on the outcome measures. In the assessment of myofascial pain, both patient-reported outcome measures (PROMs), such as the visual analogue scale (VAS), and objective measures, such as pressure pain thresholds (PPT), are commonly employed [8,20,21,22,24,25,26]. PPT is typically assessed using algometers, which provide a quantifiable and objective measure of mechanical pain sensitivity [8,24,26].

Digital pressure algometers have provided valid and reliable pressure pain threshold (PPT) measurements across diverse clinical and research populations, with intra-rater reliability consistently in the good-to-excellent range and measurement error generally low [27,28]. However, measurement properties are device-specific, and devices are not interchangeable without independent validation [29]. Therefore, newly developed algometers require independent evaluation of their validity, reliability, measurement error, and responsiveness before their implementation in clinical and research settings.

The NOD is a novel algometer designed to assess the PPT. However, before its implementation in clinical and research settings, its measurement properties must be evaluated. According to the COSMIN framework, an instrument must demonstrate adequate reliability, defined as the ability to produce consistent and reproducible results under stable conditions, and validity, defined as the degree to which it measures the construct it intends to assess [30]. When no universal gold standard exists for PPT assessment, the validity of a new instrument can be investigated by comparing its measurements with those obtained using an established reference instrument that assesses the same construct, while also checking reliability and measurement error, as interpretability depends on both [28,29,31,32]. For PPT specifically, conventional algometry is widely used as a reference method, although no universally accepted gold standard instrument has been established [31,32]. Therefore, comparison with a reference instrument provides evidence regarding the validity of the NOD, while reliability and measurement error are also essential to determine whether its measurements are consistent and interpretable.

Importantly, not all outcome measures are equally sensitive in detecting changes over time. Responsiveness, defined as the ability of an instrument to detect changes in the construct being measured over time, is a key measurement property when evaluating the effects of an intervention [30]. An outcome measure may be valid and reliable, yet fail to capture clinically relevant changes, thereby limiting its usefulness in both research and clinical practice settings. Although responsiveness is commonly investigated in clinical populations, its assessment in healthy individuals with latent trigger points may provide an initial evaluation of the NOD’s ability to detect changes under controlled experimental conditions [33,34,35]. The absence of clinically diagnosed pain reduces clinical heterogeneity [33,34,35], while the presence of latent trigger points provides a standardized myofascial condition in which temporal changes in pressure pain sensitivity can be examined.

To the best of our knowledge, the validity, reliability, and responsiveness of the NOD have not yet been assessed. Therefore, this study aimed to evaluate the validity, reliability, and responsiveness of the NOD algometer using a conventional algometer as a reference instrument for PPT assessment in healthy individuals with latent trigger points.

## 2. Materials and Methods

### 2.1. Design

A prospective methodological study with repeated measures was conducted, and participants were recruited among students at the University of Valencia between October 2024 and June 2025. The study procedures and statistical analyses followed the COSMIN recommendations for studies evaluating measurement properties. The study was approved by the Ethics Committee of the University of Valencia (Spain) (3250436) and was prospectively registered at clinicaltrials.gov (NCT06448104). All procedures complied with the principles of the Declaration of Helsinki. Participants provided their written informed consent after being informed of the study’s objective, the experimental procedure, and the potential associated risks. Furthermore, participants were instructed to avoid physical exercise for twenty-four hours before the measurements to avoid affecting the measurement results. Patient participation was voluntary. All measurements were performed by the same physiotherapist, who had previous clinical and research experience in pressure pain threshold assessment. Before participant recruitment, the evaluator completed specific familiarization sessions with the NOD device and followed a standardized assessment protocol throughout the study.

### 2.2. Participants

Forty participants were selected based on the following inclusion criteria: (1) age between 18 and 45 years; (2) have at least one identifiable latent trigger point in the muscles studied; (3) absence of spontaneous musculoskeletal pain or pain from other causes in the area to be tested; and (4) absence of absolute or relative contraindications to the application of pressure to the vastus medialis of the quadriceps due to infections, active neoplasms, allergy to materials, coagulation disorders, or the presence of implanted devices in nearby areas. All the participants completed the study.

### 2.3. Procedures

PPT measurements were obtained on the dominant leg (vastus medialis of the quadriceps) during two sessions separated by one week apart by performing PPT measurements on the primary latent trigger point identified by pain elicited by palpation. Latent trigger point was identified based on the presence of a palpable taut band and a hypersensitive point within the muscle, along with the onset of referred pain upon palpation, in accordance with established consensus criteria [10]. Since the participants were asymptomatic, the absence of spontaneous pain was ensured, and it was not necessary for them to recognize the evoked pain as a familiar sensation. In each session, PPT was assessed at four time points: baseline, immediately after the intervention, and 30 and 60 min post-intervention. At each time point, two repeated measurements were obtained for each device, resulting in eight measurements per device per session.

Concurrent validity and agreement analyses were performed using the mean of the four baseline measurements obtained across both sessions for each instrument separately. Intra-session reliability was evaluated using two repeated baseline measurements within each session. Inter-session (test–retest) reliability was assessed using the mean baseline values from each session. Responsiveness was evaluated by analyzing changes in PPT measurements across different post-intervention time points.

Concurrent validity was assessed by comparing PPT values obtained using the NOD (OT Bioelettronica Company, Turin, Italy) and a reference measure, an analogue pressure algometer (Wagner Instruments FDK 20, range 20 lb. to 0.25 lb. or 10 kg × 100 gf, Wagner Instruments, Greenwich, CT, USA). The order of application of the NOD and reference algometer was randomized at each assessment time point, following a within-subject crossover design.

Dry needling was used as an experimental pain induction procedure to generate transient changes in PPT values and evaluate the responsiveness of the NOD device. Specifically, dry needling was applied at the previously identified latent trigger point before the post-intervention measurements, as this procedure has been shown to induce short-term alterations in mechanical pain sensitivity and pressure pain thresholds. In the second session, an additional fatigue protocol was incorporated to induce broader alterations in pain sensitivity and to further assess responsiveness under combined nociceptive and fatigue-related conditions. This second condition was included to explore the ability of the NOD to detect changes in PPT under a distinct physiological context rather than to replicate the responsiveness assessment performed during the first session. From a clinical perspective, pain and fatigue frequently coexist in musculoskeletal disorders and may jointly influence pain sensitivity, supporting the inclusion of this exploratory condition.

**Figure 1:**
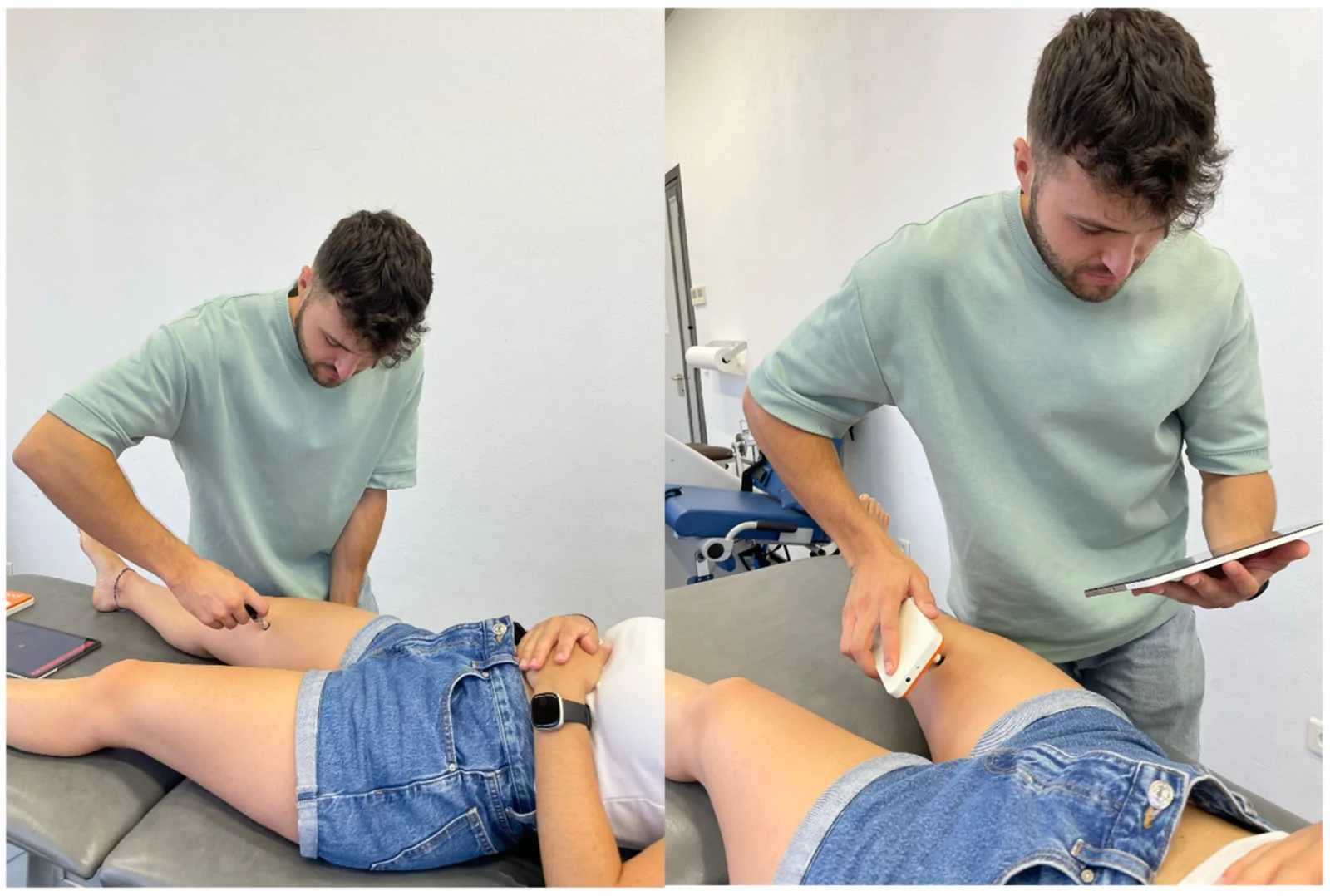
Evaluation of pain pressure thresholds using a conventional algometer (**left**) and the NOD device (**right**).

Anthropometric and demographic characteristics were collected before the tests were initiated. Two measurement sessions were conducted one week apart. In both sessions, PPTs were evaluated at the start of the session, immediately after the dry needling intervention, and 30 and 60 min after the intervention. The protocol consisted of identifying the most sensitive point on the vastus medialis muscle of the dominant leg through palpation of three consecutive points on the skin, followed by measurements at the marked point with the participant lying supine on a treatment table with the knee extended, using both a conventional analog algometer and the NOD algometer (Figure 1). During each session, all PPT measurements were performed at this identified location. For the second session, conducted one week later, the same participant position, anatomical landmarks, muscle region, and palpation procedure were used to relocate the most sensitive point before testing.

Pressure was applied perpendicularly at a constant rate of approximately 1 kg/s until the participant reported their first sensation of pain. At this point, the application of pressure was stopped, and the corresponding measurement was recorded [36]. At each assessment time point (baseline, immediately after the intervention, and 30 and 60 min post-intervention), the evaluator performed two consecutive measurements with each device, with a one-minute rest period between applications, following a randomized order of device administration. A one-minute interval was selected to minimize potential carry-over effects, local sensitization, temporal summation, and discomfort associated with repeated pressure pain threshold assessments while maintaining a feasible testing procedure.

A dry needling procedure, based on the Hong technique, was performed at the most sensitive point of the vastus medialis quadriceps muscle [37]. This technique consists of repeated needle insertion (rapid entries and exits) into the MTP, trying to obtain as many local spasm responses as possible within the patient’s tolerance [38].

In the second session, a fatigue test was conducted to combine the effects of peripheral and central fatigue with pain from dry needling, thereby assessing sensitivity to change under those conditions. Thus, prior to dry needling, the maximum voluntary isometric contraction (MVIC) of the quadriceps was assessed using a pull dynamometer (Suiff Pro, Suiff, Abrera, Spain), and participants were asked to perform a 60 s fatiguing isometric contraction at 70% of the MVIC [39].

### 2.4. Instruments

The FPK20 is an analogue pressure algometer consisting of a pressure gauge connected to a 1 cm^2^ rubber probe, with readings expressed in kg/cm^2^ (0–10 kg/cm^2^).

The NOD device is a versatile digital instrument that combines the functions of three different clinical applications: algometer, EMG biofeedback, and hand dynamometer. The NOD device is a rigid platform shaped like a rectangular prism (79 mm × 194 mm × 17 mm). When used as an algometer, a magnetized rectangle ending in a 1 cm^2^ cylindrical tip was placed on the pressure surface. The pressure detected is transmitted via Bluetooth^®^ to a smartphone or tablet, where a dedicated app allows the PPT to be displayed and recorded. The NOD device was tested for compliance with the EN 60601-1 [40] and EN 60601-1-2 standards [41].

### 2.5. Statistical Analysis

Descriptive statistics were calculated for all the variables. Continuous variables are presented as means and standard deviations (*SD*). Categorical variables are reported as frequencies and percentages. For each measurement condition (baseline, immediate post-intervention, 30 min post-intervention, and 60 min post-intervention), the mean of two repeated trials was calculated to obtain a representative value. Additionally, change scores (Δ) were computed as the difference between the baseline and each subsequent time point for both instruments.

#### 2.5.1. Concurrent Validity Analysis

For the concurrent validity analysis, the mean of the four baseline measurements obtained across the two sessions was calculated for both the NOD and algometer, providing a single representative value for each participant and instrument. Thus, a more stable estimate of each measurement was obtained by averaging across repeated trials, since the two baseline measurements were taken at rest, unaffected by the intervention or the fatigue test. The normality of the data distribution was evaluated using the Shapiro–Wilk test. As the data did not follow a normal distribution, Spearman’s rho correlation coefficient (*ρ*) was used to assess the association between the two instruments.

The magnitude of correlations was interpreted according to pain-specific recommendations, with values of 0.10, 0.30, and 0.40 representing small, moderate, and large effects, respectively [42]. Although Spearman’s rho (*ρ*) was used because of non-normality, the same interpretative framework was applied to facilitate clinical interpretation.

Bland–Altman plots were used to assess the agreement between the two instruments. In addition, the following agreement parameters were computed: limits of agreement (LoA), mean difference between tools (defined as “bias”), and the standard deviation of the differences (defined as “imprecision”). The limits of agreement were calculated as bias ± 1.96 × SD. All values were expressed as percentages relative to the algometer reference measurements. A 10% criterion was adopted from a previous validation study [43] and was used as a methodological reference rather than as a clinically established threshold for PPT measurements. A Bland–Altman plot was constructed to visually inspect agreement and identify any systematic bias or heteroscedasticity.

#### 2.5.2. Reliability Analysis

Reliability was assessed through both intra- and inter-session analyses. Intra-session reliability was evaluated separately for each session using two repeated baseline measurements obtained prior to any intervention without averaging to capture within-session variability. Inter-session (test–retest) reliability was assessed using baseline measurements obtained in two sessions, one week apart. Prior to this analysis, paired t-tests were conducted to verify the absence of systematic differences between the sessions. As no significant differences were found between the baseline measurements, the mean of the repeated baseline trials within each session was calculated and used for the reliability analysis, providing a more stable estimate of each measurement.

Intraclass correlation coefficients (ICC) were calculated using a two-way random-effects model with absolute agreement (ICC(2,1) for intra-session analyses and ICC(2,k) for inter-session analyses based on average measurements. The standard error of measurement (SEM) was computed as SEM = SD × √(1 − ICC), and the minimal detectable change (MDC) was calculated as MDC = SEM × 1.96 × √2. Additionally, the SEM and MDC were expressed as percentages of the mean to facilitate interpretation.

#### 2.5.3. Responsiveness Analysis

Responsiveness was assessed by analyzing changes in NOD measurements across different time points within each session (baseline, end of the session, 30 min, and 60 min). A repeated-measures analysis of variance (ANOVA) was performed separately for each session to evaluate the time effect. The assumption of sphericity was assessed using Mauchly’s test; if it was violated, the Greenhouse–Geisser correction was applied. Post hoc pairwise comparisons were conducted using Bonferroni adjustment to identify differences between time points. Paired *t*-tests were used as complementary analyses to quantify the magnitude and direction of change between the baseline and each time point. The normality of the change scores was evaluated using the Shapiro–Wilk test prior to the analysis. All data were normally distributed. The magnitude of change was quantified using the standardized response mean (SRM), which was calculated as the mean change divided by the standard deviation of the change scores. *Cohen’s d* was computed to estimate the effect size. Effect sizes were interpreted according to recommendations derived from pain research literature, with thresholds of 0.10, 0.30, and 0.70 indicating small, moderate, and large effects, respectively [42]. The standard errors (SE) of the mean differences are also reported.

Additionally, change scores (Δ) were computed for NOD and Algometer at the end, after 30 min, and after 60 min relative to baseline. The criterion validity of responsiveness was examined using bivariate correlations between ΔNOD and ΔAlgometer. The normality of the change scores was assessed using the Shapiro–Wilk test. As the normality assumptions were not met for several variables, Spearman’s correlation coefficient (*ρ*) was used to assess the association between changes in NOD and algometer measurements. Statistical significance was set at *p* < 0.05. The responsiveness analysis was conducted without a sham or control condition. Therefore, the observed changes cannot be attributed exclusively to the experimental interventions and may also reflect natural variability in pressure pain threshold, repeated-measurement effects, or other uncontrolled influences.

Statistical analyses were performed using coded datasets containing participant identification codes only. No participant-identifying information was available during the analysis process. All statistical analyses were performed using Jamovi software (version 2.7) [44]. The sample size was calculated using the formula for reliability studies based on confidence intervals (CI) described by Streiner and Norman [45]. With the number of instruments (*k*) equal to 2, the CI around *r* (the reliability coefficient) of 0.05, and an estimated r of 0.95, the sample size (*n*) was calculated as a minimum of 25 participants. The sample size was increased to improve the precision of reliability estimates and reduce measurement error [46].

## 3. Results

The participants’ characteristics are presented in Table 1. A total of 40 participants were included in the study (mean age: 22.0 ± 3.35 years), of whom 65% were male and 35% were female. Myofascial pain was predominantly located in the right leg (82.5%) compared with the left leg (17.5%).

In the first session, both instruments showed a progressive decrease in the pressure–pain threshold over time (Table S1). For the algometer, the mean values decreased from 4.25 ± 1.66 at baseline to 3.92 ± 2.18 immediately post-intervention, 3.72 ± 2.21 at 30 min, and 3.40 ± 2.01 at 60 min. Similarly, NOD values decreased from 4.59 ± 1.98 at baseline to 3.99 ± 1.97 immediately post-intervention, 3.68 ± 2.13 at 30 min, and 3.40 ± 1.80 at 60 min. These changes corresponded to mean reductions of −0.61, −0.91, and −1.20 for the NOD and −0.33, −0.53, and −0.85 for the algometer, respectively.

A different pattern was observed in the second session. Algometer values decreased from 4.64 ± 2.10 at baseline to 4.46 ± 2.41 immediately post-intervention, 3.99 ± 1.89 at 30 min, and 3.52 ± 1.48 at 60 min. In contrast, NOD values showed a slight increase immediately post-intervention (4.65 ± 1.85), followed by a decrease at 30 min (4.21 ± 2.08) and 60 min (3.83 ± 1.74). The corresponding changes for NOD were 0.14, −0.30, and −0.67, whereas for the algometer, they were −0.17, −0.64, and −1.12.

Overall, the combined baseline mean across both sessions (four measurements) was 4.44 ± 1.75 for the algometer and 4.55 ± 1.68 for the NOD, with a small mean difference between the instruments (0.11 ± 0.63). All pressure pain threshold values were expressed in kg/cm^2^.

**Figure 2:**
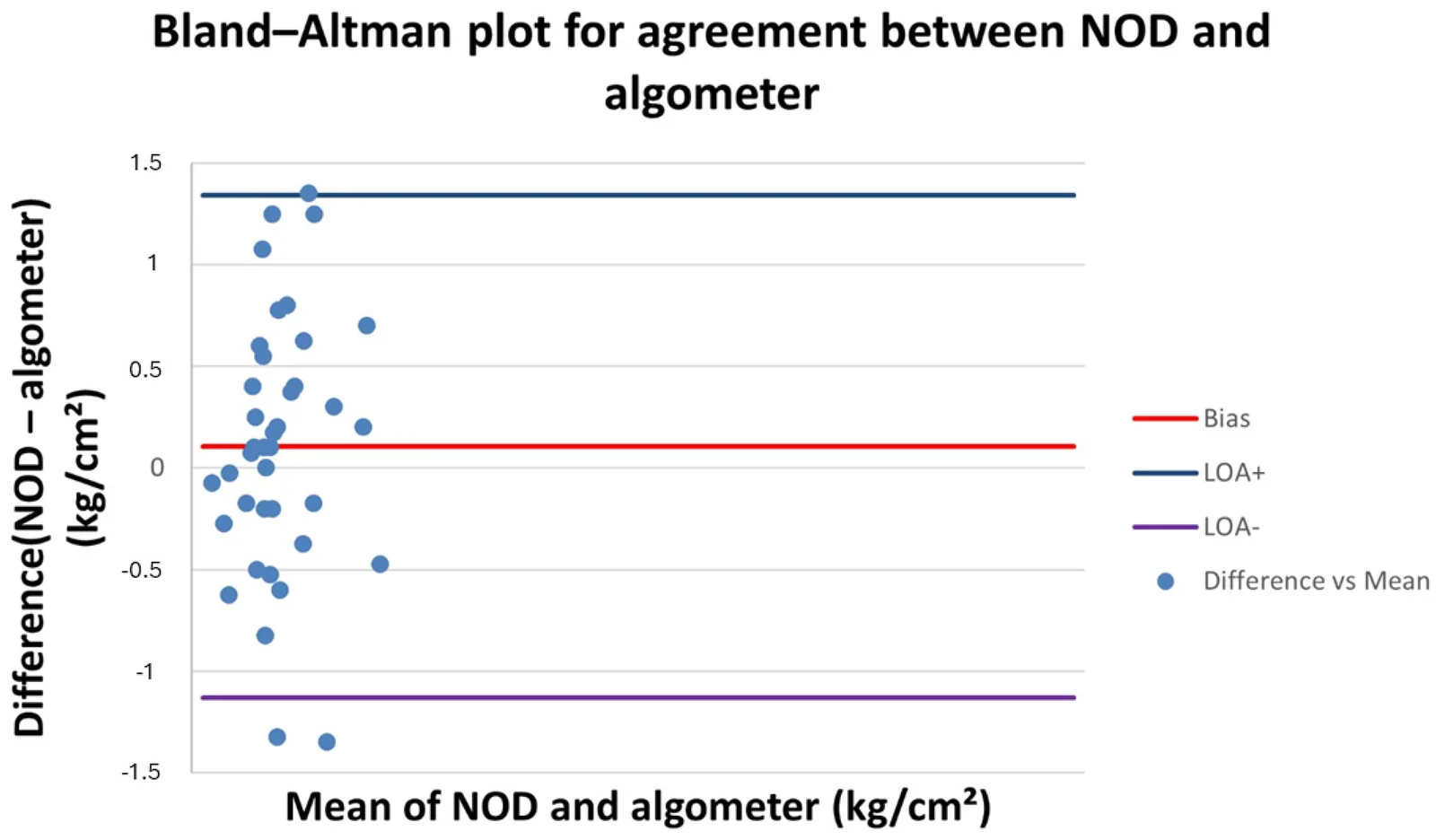
Bland–Altman plots for NOD and Algometer for pressure pain threshold (PPT).

A strong positive association (Table 2) was observed between the algometer and NOD measurements (Spearman’s ρ = 0.90, *p* < 0.001; 95% CI: 0.887–0.965). Bland–Altman analysis revealed a minimal mean difference (bias) of 0.106, corresponding to 2.39% relative to the algometer values, indicating a negligible systematic bias between the instruments despite relatively wide limits of agreement (Figure 2). However, the limits of agreement ranged from −25.45% to 30.18%, suggesting substantial variability between devices at the individual level despite the small overall bias.”

Regarding reliability (Table 3), intra-session reliability was good to excellent in both sessions (ICC = 0.825 and 0.859), with SEM values ranging from 0.63 to 0.82 (14.09–17.74%) and MDC values ranging from 1.75 to 2.26 (38.89–49.24%). Inter-session reliability was also good (ICC = 0.803; 95% CI: 0.666–0.884), with a higher measurement error (SEM = 1.05; 23.16%) and MDC (2.91; 63.96%). These findings indicate high relative reliability but moderate absolute measurement errors, particularly between sessions. These results also suggest that while the NOD provides consistent relative measurements, caution should be exercised when interpreting individual changes, given the magnitude of the measurement error.

The responsiveness results are presented in Tables S2–S6, and summarized the most important statistics in Table 4. Repeated-measures ANOVA revealed a significant effect of time in both sessions (Session 1: *F*(2.08,81.18) = 13.40, *p* < 0.001; Session 2: *F*(2.62,102.21) = 9.48, *p* < 0.001). Detailed ANOVA, sphericity tests, and post-hoc comparisons are provided in Supplementary Tables S2–S4.

In the first session, post hoc analyses revealed significant reductions in NOD values at all post-intervention time points compared to baseline (*p* ≤ 0.005), with no differences between the immediate post and 30 min time points, but a significant additional reduction at 60 min compared to the immediate post time point (*p* = 0.024). The magnitude of change increased over time, with SRM values ranging from −0.57 to −0.74 and moderate-to-large effect sizes (*Cohen’s d* = 0.57–0.73) (Table 4, Tables S4 and S5).

In the second session, no significant differences were observed between the baseline and either the immediate post-intervention or the 30 min post-intervention. However, a significant reduction was observed at 60 min compared to the baseline (*p* = 0.007). Post-hoc comparisons also revealed significant differences between the immediate post-and later time points. The magnitude of change was smaller than that in the first session, with SRM values ranging from 0.17 to −0.56 and small-to-moderate effect sizes (Table 4, Tables S4 and S5).

The association between changes in the NOD and algometer measurements is presented in Table S6. In the first session, significant poor-to-moderate positive correlations were observed at all time points, with Spearman’s ρ values of 0.342 (*p* = 0.031) immediately post-intervention, 0.570 (*p* < 0.001) at 30 min, and 0.696 (*p* < 0.001) at 60 min. These results indicate a consistent and increasing agreement between the two instruments in detecting changes over time.

In the second session, the patterns were less consistent. A significant poor correlation was observed immediately post-intervention (*ρ* = 0.405, *p* = 0.009), whereas no significant association was found at 30 min (*ρ* = 0.066, *p* = 0.687). At 60 min, a significant but poor correlation was identified (*ρ* = 0.364, *p* = 0.021). Overall, responsiveness was demonstrated under the first experimental condition, whereas agreement in detecting change was less consistent during the second condition incorporating fatigue. These findings suggest that the ability of the NOD to track changes may depend on the physiological context in which pressure pain threshold is modified.

## 4. Discussion

This study aimed to examine the validity, reliability, and responsiveness of the NOD device for measuring the PPT, thereby providing an accessible digital tool for the psychophysical measurement of pain. Our results indicate that the NOD is a valid and reliable tool for measuring PPT at latent trigger points in healthy participants. Specifically, concurrent validity revealed a high correlation with validated reference instrument and a small average bias, although the limits of agreement were relatively wide. Both the reliability and the measurement error (SEM) were found to be adequate; however, given the magnitude of the SEM, the results should be interpreted with caution. Finally, the responsiveness of the NOD demonstrated an overall capacity to detect changes over time, particularly 60 min after the intervention in the first session. A clear time-dependent reduction was observed, with increasing effect sizes over time, suggesting a progressive response detectable by the instrument. The moderate SRM and Cohen’s d values further support the ability of the NOD to detect changes over time. In contrast, the second session showed more limited responsiveness, with only delayed changes reaching statistical significance. This difference may be explained by the combined influence of fatigue and intervention, which potentially attenuates the immediate response or increases variability in the measurements. The lower SRM values observed in this session suggest a reduced sensitivity to change under these conditions. Overall, these results indicate that the NOD shows an ability to detect changes in the PPT over time, particularly in conditions where a clear physiological change is expected, although its sensitivity may be influenced by the nature of the intervention and variability introduced by experimental conditions. Caution is warranted when interpreting individual changes in clinical practice, since PPT is highly individualized, dynamic, and variable in nature.

A strong correlation was observed between the NOD and the reference algometer (ρ = 0.90), indicating that both devices ranked participants similarly across the measurement range. However, agreement analysis provides complementary information. Although the mean bias was small (2.39%), the limits of agreement ranged from approximately −25% to +30%, indicating that individual measurements may differ substantially between devices. Therefore, the present findings support strong association and limited systematic bias, but they do not necessarily support complete interchangeability between instruments.

While various studies have indicated that manual palpation is a reliable method for identifying MPS, certain limitations inherent in the diagnostic criteria have been highlighted [12]. Consequently, the PPT has been recommended as a more accurate and valid method for evaluating pain sensitivity in myofascial pain syndrome [25]. Given that increased sensitivity to PPT has been found to be associated with musculoskeletal symptoms [47], such assessment is particularly valuable for identifying an individual’s pain psychophysical profile.

Our findings are consistent with previous evidence regarding the evaluation of PPT. Mallioux et al. [48] found that intrasession assessment of PPT in pain-free participants yielded a significant change ranging from 28.71 to 50.56 kPa across the sites tested. Similarly, the authors found adequate reliability, as measured by ICC and SEM. Moreover, Koh et al. [49] showed high inter-rater and intra-rater reliability (ICC > 0.90) and MDD values (0.88 kg/cm^2^ in the first session and 1.24 kg/cm^2^ in the second evaluation) obtained using this PPT algometry on pain-free participants, with two evaluators over two consecutive days. NOD showed an MDC of 2.26 (kg/cm^2^) in the first session and 1.75 (kg/cm^2^) in the second session, with an inter-session value of 2.91 (kg/cm^2^). Notably, Bellosta-López et al. [50] demonstrated proper reliability of the PPT assessment in the medium term (2 weeks) and long term (6 months), which strengthens the capacity of this procedure to assess the pain-sensory profile over time.

An important consideration when interpreting the responsiveness results is that the two sessions did not evaluate identical experimental conditions. The first session assessed changes following dry needling alone, whereas the second session combined dry needling with a fatigue-inducing protocol. Accordingly, the second session should not be interpreted as a direct replication of the first session or as an independent validation of responsiveness. Rather, it provided an exploratory assessment of the instrument’s performance under a distinct physiological condition that may involve additional peripheral and central mechanisms influencing pain sensitivity. The reduced agreement observed during the second session may therefore reflect differences in the physiological response rather than solely limitations of the instrument.

Moving from the lab to clinical practice, evaluating pain-sensory profiles in a research setting has enabled researchers to classify patients by pain phenotype, establish a prognosis, and potentially predict their response to specific treatments. Quantitative sensory testing (QST), which uses controlled painful stimuli to assess various aspects of pain sensitivity, has been widely used to evaluate pain psychophysical testing and the effects of various therapeutic interventions. Both manual therapy and physical therapy modalities and interventions have been found to increase PPT values in subjects with MPS; however, it must be considered that the duration of the increase in PPT following an intervention has not been reported [51]. While previous studies have shown a strong correlation between PPT scores, it has been suggested that a brief battery of tests consisting of two PPT scores, along with other psychophysical tests, may be sufficient to provide meaningful information about the pain-sensory profile and to assess the hypoalgesic effects of physiotherapy interventions [50]. Therefore, the NOD may provide a valid, reliable and accessible method for assessing the pain-sensory profile in physiotherapy. However, the relatively large SEM and MDC values suggest that caution is required when interpreting individual longitudinal changes, particularly when the magnitude of change is small. Furthermore, future studies are needed to confirm the NOD’s usefulness in clinical pain profiles.

The added value of the NOD lies in its multifunctional wireless platform, which integrates dynamometry, algometry, biofeedback, and EMG into a single compact device. This novel instrument was designed to allow both clinical and research evaluations of strength, pain threshold, and muscle activity with precision and user-friendliness in a single device. This shows a promising avenue for multidimensional digital physiotherapy assessment.

### Strengths, Limitations and Future Research

This is the first study to evaluate the validity, reliability, and responsiveness of the NOD portable device for measuring PPT, offering a multifunctional device for digital assessment in physiotherapy. Pain psychometric evaluation of PPT across multiple time series also revealed the value of the instrument in sensitively tracking changes over time following the application of a physiotherapy intervention, such as dry needling. This suggests the potential of this novel device for assessing and monitoring hypoalgesic effects across various physiotherapy approaches.

Despite its relevant contributions, this study has several limitations that should be considered. First, considering that this study was conducted in a healthy population, the current results are not generalizable to other populations, such as those with chronic pain conditions. Second, it is acknowledged that several factors, such as race/ethnicity, sex, age, and pain condition, could influence pain sensitivity [52,53,54,55] have not been analyzed. However, considering that we assessed the reliability of pain sensitivity in a cohort of participants with no prior pain, rather than pain sensitivity itself, the impact of these pain-sensory profiles on our results remains limited. Third, the limited time-tracking of this study prevents drawing conclusions about the long-term reliability of the device. Although digital algometers can improve the accuracy of PPT (i.e., they can increase force at a constant rate), the manual algometer—which has demonstrated excellent inter-rater and intra-rater reliability—remains the validated reference instrument for this procedure. Fourth, intra-rater reliability was not measured in this study. This limits the reliability of the reproducibility assessment of the NOD device. Also, although measurement site localization was standardized using the same evaluator, anatomical region, participant position, and palpation procedure across sessions, no permanent skin marking or coordinate-based anatomical mapping was employed. Therefore, a small amount of between-session variability related to point relocation cannot be completely ruled out. Clinicians must consider this when using the PPTS in clinical practice. Finally, given that the measuring error between sessions and the MDC are relatively high, caution is encouraged when interpreting individual changes in clinical practice. Neither participant nor assessor blinding was feasible because of the visible and operational differences between the devices being compared. Although device order was randomized and a standardized assessment protocol was used, the absence of blinding may have introduced a degree of measurement bias and should be considered when interpreting the results. Additionally, responsiveness was evaluated without a sham or control condition. Consequently, although the interventions were intended to induce changes in pressure pain threshold, the observed responses may also reflect natural variability in pressure pain sensitivity, repeated-testing effects, or measurement-related factors. Therefore, causal inferences regarding the source of the observed changes should be made with caution.

Finally, future studies should evaluate the reliability of the NOD device over time and across different clinical and sensory pain profiles to ensure the generalizability of the measurements obtained using the instrument.

## 5. Conclusions

The NOD exhibited a strong association with a conventional reference algometer and good relative reliability under standardized experimental conditions in healthy participants with latent trigger points. The device was also able to detect experimentally induced changes in pressure pain threshold over time. However, the relatively wide limits of agreement indicate that interchangeability with conventional algometers cannot yet be assumed, despite the small average bias observed between devices. Furthermore, responsiveness was evaluated under experimental conditions in healthy individuals and should not be interpreted as evidence of responsiveness to clinically meaningful change. Further studies in musculoskeletal pain populations are needed to establish the clinical applicability of the NOD.

## Figures and Tables

**Table 1:** Participants’ characteristics.

Outcome	Mean (SD)	Outcome	N (%)
**Age (years)**	22.00 (3.35)	**Sex**	
		Male	26 (65%)
		Female	14 (35%)
**Height (cm)**	174.00 (10.40)	**Leg assessed**	
**Weight (kg)**	72.20 (13.30)	Right	33 (82.50%)
		Left	7 (17.50%)

**Table 2:** Concurrent validity between Algometer and NOD to measure pressure pain threshold in the latent trigger point of the vastus medialis muscle of the dominant leg.

Algometer (*n* = 40) Mean (SD) [min, max]	NOD (*n* = 40) Mean (SD) [min, max]	Shapiro–Wilk (W, *p*)	Spearman’s Rho (*ρ, p*) [95%CI]	Lo A−	Lo A+	Mean Difference (%) [Shapiro–Wilk’s *p*]	SD Difference (%)
**4.44 (kg/cm^2^) (1.75) [1.55, 9.63]**	4.55 (kg/cm^2^) (1.68) [1.27, 8.88]	**Algometer’s** 0.924, 0.010 **NOD’s** 0.956, 0.119	0.900 *** (<0.001) [0.887, 0.965]	−1.13 (kg/cm^2^) (−25.45%)	1.34 (kg/cm^2^) (30.18%)	0.106 (kg/cm^2^) (2.39%) [0.727]	0.629 (kg/cm^2^) (14.17%)

SD: standard deviation; LoA: limit of agreement. *** *p* < 0.001.

**Table 3:** Reliability of the NOD in measuring the pressure pain threshold in the latent trigger point of the vastus medialis muscle of the dominant leg.

Reliability	Retest (SD)	ICC (95% CI)	SEM (SEM%)	MDC (MDC%)
**Intra-session (First session)**	4.59 (kg/cm^2^) (1.98)	0.825 (0.704, 0.897)	0.82 (kg/cm^2^) (17.74%)	2.26 (kg/cm^2^) (49.24%)
**Intra-session (Second session)**	4.50 (kg/cm^2^) (1.70)	0.859 (0.764, 0.926)	0.63 (kg/cm^2^) (14.09%)	1.75 (kg/cm^2^) (38.89%)
**Inter-session**	4.55 (kg/cm^2^) (1.68)	0.803 (0.666, 0.884)	1.05 (kg/cm^2^) (23.16%)	2.91 (kg/cm^2^) (63.96%)

SD: standard deviation; ICC: intraclass correlation coefficient; SEM: standard error of measurement; MDC: minimum detectable change.

**Table 4:** Responsiveness of the NOD device across experimental conditions.

Session	Time Comparison	Mean Change (kg/cm^2^)	SRM	*Cohen’s d*	*p*-Value
Session 1 (Dry Needling)	Baseline vs. End	−0.61	−0.57	0.57	<0.001 ***
	Baseline vs. 30 min	−0.91	−0.62	0.62	<0.001 ***
	Baseline vs. 60 min	−1.20	−0.74	0.73	<0.001 ***
Session 2 (Fatigue + Dry Needling)	Baseline vs. End	0.14	0.17	0.17	0.303
	Baseline vs. 30 min	−0.30	−0.29	0.29	0.078
	Baseline vs. 60 min	−0.67	−0.56	0.56	0.001 ***

SRM: standardized response means; Effect size: *Cohen’s d*. SRM values retain the direction of change, whereas *Cohen’s d* values are presented as absolute magnitudes of effect. *** *p* < 0.001.

## Data Availability

The datasets generated and analyzed during the current study are not publicly available but are available from the corresponding author upon reasonable request.

## Supplementary Materials

The following supporting information can be downloaded at https://www.mdpi.com/article/10.3390/jcm15176687/s1, Table S1. Descriptive pressure pain threshold values obtained with the reference algometer and the NOD device across all assessment time points and sessions; Table S2. Repeated-measures ANOVA for responsiveness analyses; Table S3. Sphericity tests for responsiveness analyses; Table S4. Post-hoc pairwise comparisons between assessment time points; Table S5. Effect Size and SRM; Table S6. Correlations between changes in NOD and reference algometer measurements.
