# Supplementary material for: Validity, Reliability, and Responsiveness of a New Device for Measuring Pressure Pain Thresholds
Source: J Clin Med. 2026 Aug 28;15(17):6687. doi: 10.3390/jcm15176687 (PMC13565762; doi:10.3390/jcm15176687)
Supplement: Supplementary file 1 [file jcm-15-06687-s001.zip › jcm-4499774-supplementary.pdf]

Table S1. Descriptive pressure pain threshold values obtained with the reference algometer and the NOD device across all assessment time points and sessions.

Outcome	Mean (SD)	Outcome	N (%)
**Algometer, First Session**			
**Baseline (1) (kg/cm²)**	4.49 (1.85)	Baseline (2) (kg/cm²)	4.01 (1.57)
**Mean baseline (kg/cm²)**	4.25 (1.66)	At the end of the session (1) (kg/cm²)	3.95 (2.12)
**At the end of the session (2) (kg/cm²)**	3.90 (2.29)	Mean at the end of the session (kg/cm²)	3.92 (2.18)
**After 30 minutes (1) (kg/cm²)**	3.87 (2.29)	After 30 minutes (2) (kg/cm²)	3.58 (2.18)
**Mean after 30 minutes (kg/cm²)**	3.72 (2.21)	After 60 minutes (1) (kg/cm²)	3.48 (2.11)
**After 60 minutes (2) (kg/cm²)**	3.31 (1.97)	Mean after 60 minutes (kg/cm²)	3.40 (2.01)
**NOD, First Session**			
**Baseline (1) (kg/cm²)**	4.83 (2.30)	Baseline (2) (kg/cm²)	4.36 (1.78)
**Mean baseline (kg/cm²)**	4.59 (1.98)	At the end of the session (1) (kg/cm²)	4.24 (2.35)
**At the end of the session (2) (kg/cm²)**	3.73 (1.70)	Mean at the end of the session (kg/cm²)	3.99 (1.97)
**After 30 minutes (1) (kg/cm²)**	3.83 (2.37)	After 30 minutes (2) (kg/cm²)	3.53 (2.03)
**Mean after 30 minutes (kg/cm²)**	3.68 (2.13)	After 60 minutes (1) (kg/cm²)	3.45 (1.95)
**After 60 minutes (2) (kg/cm²)**	3.35 (1.78)	Mean after 60 minutes (kg/cm²)	3.40 (1.80)
**Algometer, Second Session**			
**Baseline (1) (kg/cm²)**	4.95 (2.49)	Baseline (2) (kg/cm²)	4.33 (1.84)
**Mean baseline (kg/cm²)**	4.64 (2.10)	At the end of the session (1) (kg/cm²)	4.58 (2.54)
**At the end of the session (2) (kg/cm²)**	4.34 (2.34)	Mean at the end of the session (kg/cm²)	4.46 (2.41)

**After 30 minutes (1) (kg/cm²)**	4.04 (1.94)	After 30 minutes (2) (kg/cm²)	3.95 (1.92)
**Mean after 30 minutes (kg/cm²)**	3.99 (1.89)	After 60 minutes (1) (kg/cm²)	3.47 (1.59)
**After 60 minutes (2) (kg/cm²)**	3.57 (1.48)	Mean after 60 minutes (kg/cm²)	3.52 (1.48)
**NOD, Second Session**			
**Baseline (1) (kg/cm²)**	4.66 (1.70)	Baseline (2) (kg/cm²)	4.35 (1.81)
**Mean baseline (kg/cm²)**	4.50 (1.70)	At the end of the session (1) (kg/cm²)	4.56 (1.89)
**At the end of the session (2) (kg/cm²)**	4.74 (1.97)	Mean at the end of the session (kg/cm²)	4.65 (1.85)
**After 30 minutes (1) (kg/cm²)**	4.17 (2.13)	After 30 minutes (2) (kg/cm²)	4.24 (2.15)
**Mean after 30 minutes (kg/cm²)**	4.21 (2.08)	After 60 minutes (1) (kg/cm²)	3.89 (1.80)
**After 60 minutes (2) (kg/cm²)**	3.77 (1.91)	Mean after 60 minutes (kg/cm²)	3.83 (1.74)
**Algometer and NOD means and increases (Δ)**			
**Algometer mean baseline first and second session (four measurements) (kg/cm²)**	4.44 (1.75)	NOD mean baseline first and second session (four measurements) (kg/cm²)	4.55 (1.68)
**NOD Δ Baseline- end of the session, first session (kg/cm²)**	-0.61 (1.06)	NOD Δ Baseline- after 30 minutes, first session (kg/cm²)	-0.91 (1.48)
**NOD Δ Baseline- after 60 minutes, first session (kg/cm²)**	-1.20 (1.63)	NOD Δ Baseline- end of the session, second session (kg/cm²)	0.14 (0.86)
**NOD Δ Baseline- after 30 minutes, second session (kg/cm²)**	-0.30 (1.05)	NOD Δ Baseline- after 60 minutes, second session (kg/cm²)	-0.67 (1.21)
**Algometer Δ Baseline- end of the session, first session (kg/cm²)**	-0.33 (1.59)	Algometer Δ Baseline- after 30 minutes, first session (kg/cm²)	-0.53 (1.33)
**Algometer Δ Baseline- after 60 minutes, first session (kg/cm²)**	-0.85 (1.39)	Algometer Δ Baseline- end of the session, second session (kg/cm²)	-0.17 (1.50)
**Algometer Δ Baseline- after 30 minutes, second session (kg/cm²)**	-0.64 (1.05)	Δ Baseline- after 60 minutes, second session (kg/cm²)	-1.12 (1.35)
**Algometer + NOD mean (kg/cm²)**	4.50 (1.69)	Mean Difference (NOD – Algometer) (kg/cm²)	0.11 (0.63)

Table S2. Repeated-measures ANOVA for responsiveness analyses.

Effect	F (df1, df2)	P	η^2p
**Time (Greenhouse–Geisser) – First session**	F(2.08, 81.18) = 13.40	<.001	0.255
**Time (Greenhouse–Geisser) – Second session**	F(2.62, 102.21) = 9.48	<.001	0.196

Table S3. Sphericity tests for responsiveness analyses.

Effect	Mauchly's W	p	Greenhouse–Geisser ε	Huynh–Feldt ε
**Time – First session**	0.527	<.001	0.694	0.734
**Time – Second session**	0.788	.110	0.874	0.942

Table S4. Post-hoc pairwise comparisons between assessment time points.

Comparison			Comparison				
**Time**	**Time**	**Time**	**Time**	**Time**	**Time**	**Time**	**Time**
			**First Session**				
**Baseline**	-	At the end	0.608	0.168	39.0	3.61	.005
	-	30 min	0.911	0.234	39.0	3.90	.002
	-	60 min	1.195	0.258	39.0	4.63	<.001
**At the end**	-	30 min	0.304	0.156	39.0	1.95	.353
	-	60 min	0.587	0.192	39.0	3.06	.024
**30 min**	-	60 min	0.284	0.157	39.0	1.81	.469
			**Second Session**				
**Baseline**	-	At the end	-0.141	0.135	39.0	-1.04	1.000
	-	30 min	0.300	0.166	39.0	1.81	.470
	-	60 min	0.674	0.191	39.0	3.52	.007
**At the end**	-	30 min	0.441	0.134	39.0	3.29	.013
	-	60 min	0.815	0.181	39.0	4.51	<.001
**30 min**	-	60 min	0.374	0.178	39.0	2.10	.251

Table S5. Effect Size and SRM.

Time	N	Mean (SD)	Comparison	Mean change (SD, SE)	SRM (Mean change/SD)	Cohen's d	p
**Session 1**							
**Baseline**	40	4.59 (1.98)	–	–	–	–	–
**At the end**	40	3.99 (1.97)	Baseline – At the end	-0.61 (1.06, 0.17)	-0.57	0.57	<.001
**After 30 min**	40	3.68 (2.13)	Baseline – After 30 min	-0.91 (1.48, 0.23)	-0.62	0.62	<.001
**After 60 min**	40	3.40 (1.80)	Baseline – After 60 min	-1.20 (1.63, 0.26)	-0.74	0.73	<.001
**Session 2**							
**Baseline**	40	4.50 (1.70)	–	–	–	–	–
**At the end**	40	4.65 (1.85)	Baseline – At the end	0.14 (0.86, 0.14)	0.17	-0.17	.303
**After 30 min**	40	4.21 (2.08)	Baseline – After 30 min	-0.30 (1.05, 0.17)	-0.29	0.29	.078
**After 60 min**	40	3.83 (1.74)	Baseline – After 60 min	-0.67 (1.21, 0.19)	-0.56	0.56	.001

Table S6. Correlations between changes in NOD and reference algometer measurements.

Outcomes – First session	Spearman's ϱ	p	Outcomes – Second session	Spearman's ϱ	p
**ΔNOD (At the end) – ΔAlgometer (At the end)**	0.342	.031	ΔNOD (At the end) – ΔAlgometer (At the end)	0.405	.009
**ΔNOD (30 min) – ΔAlgometer (30 min)**	0.570	<.001	ΔNOD (30 min) – ΔAlgometer (30 min)	0.066	.687
**ΔNOD (60 min) – ΔAlgometer (60 min)**	0.696	<.001	ΔNOD (60 min) – ΔAlgometer (60 min)	0.364	.021
